# Optimizing Intubation Prediction in Pneumonia Patients: A Systematic Review and Meta‐Analysis of Machine Learning Algorithms

**DOI:** 10.1155/pm/6670267

**Published:** 2026-03-18

**Authors:** Elham Abdoli, Pooya Eini, Sajjad Farashi, Maryam Farhadian

**Affiliations:** ^1^ Infectious Disease Research Center, Hamadan University of Medical Sciences, Hamadan, Iran, umsha.ac.ir; ^2^ Neurophysiology Research Center, Institute of Neuroscience and Mental Health, Avicenna Health Research Institute, Hamadan University of Medical Sciences, Hamadan, Iran, umsha.ac.ir; ^3^ Department of Biostatistics, School of Public Health, Research Center for Health Sciences, Hamadan University of Medical Sciences, Hamadan, Iran, umsha.ac.ir

**Keywords:** COVID-19, endotracheal intubation, influenza, machine learning, pneumonia

## Abstract

**Background:**

Pneumonia, including influenza, COVID‐19, and community‐acquired pneumonia, is a major global health burden associated with high morbidity, mortality, and frequent progression to respiratory failure requiring intubation. Early identification of patients at risk of endotracheal intubation is essential to improve outcomes and optimize ICU resource allocation, yet existing prognostic tools remain limited in predicting this need. This study evaluated the performance of machine learning (ML) algorithms in predicting endotracheal intubation among patients with pneumonia during hospital stay.

**Methods:**

We systematically searched five databases to evaluate the diagnostic accuracy of ML models. Pooled estimates of area under the receiver operating characteristic curve (AUROC), sensitivity, and specificity were calculated. Subgroup analysis and meta‐regression were conducted. Risk of bias was assessed using PROBAST+AI and certainty of evidence with GRADE.

**Results:**

This systematic review of 34 studies (26 in meta‐analysis) included 195,214 pneumonia patients. The pooled AUROC was 0.79 (95% CI: 0.75–0.82), with sensitivity of 0.74 (95% CI: 0.61–0.84), specificity of 0.71 (95% CI: 0.50–0.86), and a DOR of 7 (95% CI: 2–20), indicating moderate diagnostic accuracy. Heterogeneity was substantial across analyses (I^2^ = 90.45*%* for sensitivity and 94.58% for specificity). Risk of bias was lowest in development (59%) and highest in application domains (41% high risk). Despite a nonsignificant Deeks′ test (*p* = 0.252), the funnel plot suggests selective publication of positive results, likely inflating the pooled AUROC. GRADE rated the evidence as moderate to low due to heterogeneity and imprecision.

**Conclusion:**

ML algorithms demonstrate a modest and highly variable accuracy in predicting the need for endotracheal intubation among pneumonia patients. High heterogeneity and methodological variability highlight the need for standardized ML approaches before clinical adoption.

## 1. Introduction

Pneumonia, encompassing various forms such as influenza, COVID‐19, and community‐acquired pneumonia (CAP), represents a significant global health challenge due to its high prevalence and associated morbidity and mortality [1]. Influenza epidemics contribute substantially to global mortality and morbidity annually, driven by the virus′s adaptability, intrinsic virulence, and population susceptibility, with estimates suggesting millions of hospital admissions and nearly half a million deaths each year [2]. The COVID‐19 pandemic further amplified this burden, overwhelming hospital services with a surge of patients, particularly those requiring invasive mechanical ventilation (IMV) due to acute respiratory distress syndrome (ARDS), while straining resources such as ventilators and healthcare personnel [3]. Similarly, CAP remains a leading cause of acute hypoxemic respiratory failure necessitating hospitalization, with delayed intensive care unit (ICU) transfers linked to poor outcomes [4]. The unprecedented scale of the COVID‐19 pandemic underscores the critical need for effective resource management, highlighting the importance of identifying at‐risk patients early to optimize care delivery across these pneumonia types [5].

The need for intubation is a pivotal concern in managing severe pneumonia cases, as it often signals progression to respiratory failure, a complication that can range widely in incidence depending on definition and context [6, 7]. In COVID‐19, the decision to intubate remains controversial, with evidence suggesting early IMV may improve outcomes, yet observational biases complicate these findings [8]. For CAP, current prognostic tools like the pneumonia severity index (PSI) excel at mortality prediction but fall short in anticipating the need for advanced respiratory support, such as intubation, during hospitalization [9]. Accurate prediction of intubation risk is crucial, as it enables timely interventions such as intensified monitoring or pre‐emptive respiratory support potentially reducing mortality and optimizing ICU resource allocation, particularly in resource‐constrained settings during pandemics.

Artificial intelligence (AI) and machine learning (ML) have emerged as transformative tools in healthcare, offering enhanced capabilities for risk stratification and prognosis by analyzing complex, multidimensional clinical data [10, 11]. These technologies have been increasingly utilized to improve diagnosis, treatment, and outcome prediction in conditions like COVID‐19, where traditional statistical methods struggle with collinearity and variable selection [12]. ML approaches, including self‐explainable techniques and regularization models, provide rigorous insights into clinical questions where randomized trials are impractical, paving the way for personalized medicine [13]. In the context of pneumonia, ML has shown promise in developing predictive tools, such as for respiratory deterioration in CAP, suggesting its potential to forecast intubation needs by leveraging diverse input variables like demographics, laboratory results, and clinical scores [14]. Current ML models, as reported in the literature, are not sufficiently reliable or standardized for clinical deployment.

This systematic review and meta‐analysis are aimed at evaluating the diagnostic accuracy of ML algorithms in predicting the need for intubation in pneumonia patients, synthesizing evidence from a large cohort across different pneumonia types. Given the global health impact of pneumonia and the critical role of timely intubation in patient outcomes, this study addresses a pressing clinical need by assessing the performance, variability, and applicability of these ML models. The findings may help guide the integration of AI‐driven systems into clinical practice, providing a foundation for standardized approaches to enhance resource allocation and patient care, particularly in postpandemic settings.

## 2. Methods

### 2.1. Study Design and Registration

We conducted a systematic review and meta‐analysis in accordance with the Preferred Reporting Items for Systematic Reviews and Meta‐Analyses (PRISMA) guidelines. The protocol was registered with PROSPERO (No: CRD420251064236). All amendments to the protocol were documented and reported in the final manuscript, and the full PRISMA checklist is available in the supporting information [15].

### 2.2. Information Sources

We searched five electronic databases: PubMed, Scopus, Web of Science, EBSCO, and Embase. We screened relevant Google Scholar records to capture gray literature, performed cross‐database deduplication against other sources, and included 200 unique records in the final screening flow.

### 2.3. Search Strategy

A comprehensive search strategy was developed to identify studies on ML for predicting intubation in pneumonia, COVID‐19, or influenza patients, targeting title, abstract, and keywords fields. The full search syntax for each database is provided in Table S2. Searches were conducted until 30 May 2025 by two reviewers, with results exported to EndNote for deduplication.

### 2.4. Eligibility Criteria

Eligibility criteria were defined using the Population, Intervention, Comparator, Outcome (PICO) framework [16] and are detailed in Table S1. We included studies involving adult patients (aged ≥ 18 years) diagnosed with pneumonia (community‐acquired, hospital‐acquired, or ventilator‐associated), COVID‐19, or influenza, admitted to hospital settings, including emergency departments, wards, or ICUs. Studies on ML algorithms, including AI and deep learning models used to predict intratracheal intubation, endotracheal intubation, respiratory insufficiency, respiratory failure, respiratory depression, or ventilatory depression, were eligible. Primary outcomes included performance metrics such as area under the receiver operating characteristic curve (AUROC), sensitivity, specificity, and accuracy, whereas secondary outcomes encompassed key predictors, model type performance, and clinical utility. Observational studies, clinical trials, and diagnostic/prognostic studies published in English until 30 May 2025 were included, with no geographical restrictions. Case reports, case series, editorials, narrative reviews, pediatric populations, nonhospitalized patients, and studies on nonrespiratory diagnoses were excluded.

### 2.5. Study Selection

Two reviewers independently screened titles and abstracts against eligibility criteria using the EndNote tool. Full‐text articles of potentially eligible studies were retrieved and assessed. Discrepancies were resolved through discussion or consultation with a third reviewer.

### 2.6. Data Extraction

Two reviewers independently extracted data using an Excel form, capturing study characteristics (author, year, country, design, sample size, and setting), population details (age, sex, diagnosis, and comorbidities), intervention specifics (model type, input features like SpO2, CRP, or radiology reports, training/validation methods), outcomes (AUROC, sensitivity, specificity, accuracy with 95% confidence intervals), predictors, and quality assessment details. Discrepancies were resolved by consensus or a third reviewer.

In accordance with the PRISMA‐DTA guidelines, we explicitly defined the index test, reference standard, and threshold handling procedures. The index test in all included studies was a ML‐based model developed to predict intubation in patients with pneumonia. Model architectures and input modalities (electronic health record [EHR] features, laboratory variables, or imaging data) are summarized in Table 1. In all included studies, the reference standard for ground‐truth intubation was based on electronic clinical or hospital records, as reported by the original authors, documenting confirmed instances of mechanical ventilation or endotracheal intubation. When multiple prediction thresholds were reported, we extracted the best‐performing threshold (as defined by the original authors). All studies defined intubation as a binary outcome (yes/no), and none relied on reader‐assigned scores or variable cutoffs.

**Table 1:** Detailed characteristics of included studies.

First author/year	Type of study	Population/country	Validation	Pneumonia type	Sample size	Intubation rate	Features	Intubation from admission	Mean age	Male %	All ML‐algorithms	Best predictor algorithm	Important features
Burdick et al. 2020 [17]	RCT	United States	Fivefold cross‐validation	COVID‐19	197	5%	15 clinical/lab data	24 h	—	51.30%	Logistic regression	Logistic regression	—
Ferrari et al. 2020 [18]	Cohort	Italy	10‐fold cross‐validation	COVID‐19	267	56%	90 clinical/lab data	48 h	—	—	LightGBM	LightGBM	Dyspnoea, PCR, pCO2, LDH, НСОЗ, CK, age, creatinine, AST‐GPT, magnesium, INR, heart rate, D‐dimer, hemoglobin, platelets, red blood cells, lymphocytes (%), respiratory rate (RR), chronic kidney insufficiency, and pH
Lundon et al. 2020 [19]	Cohort	United States	Fivefold cross‐validation	COVID‐19	484	7%	25 clinical/lab data	During hospital stay	71.8	93.30%	Decision tree	Decision tree	Identified age, sex, temperature on admission, systolic blood pressure on admission, RR on admission, and lowest oxygen saturation
Aljouie et al. 2021 [20]	Cohort	Saudi Arabia	Fivefold cross‐validation	COVID‐19	1508	12%	34 clinical/lab/imaging data	72 h	53.42 ± 16.63	55.20%	Support vector machine, random forest, logistic regression, and XGBoost	Support vector machines	Age, gender, CXR findings, and CBC
Arvind et al. 2021 [21]	Cohort	United States	Fivefold cross‐validation	COVID‐19	4087	11%	23 clinical/lab data	72 h	58.6 ± 21.90	34.60%	Random forest	Random forest	Hypertension with complications, RR, pH, temperature, oxygen saturation, platelet count, and arterial CO2
Bolourani et al. 2021 [22]	Cohort	United States	10‐fold cross‐validation	COVID‐19	11,525	8%	34 clinical/lab data	48 h	65	58%	XGBoost, XGBoost +SMOTEENN, and logistic regression	XGBoost	Type of oxygen delivery used in the emergency department, patient age, Emergency Severity Index level, RR, serum lactate, and demographic characteristics
Campbell et al. 2021 [23]	Cohort	United States	External validation	COVID‐19	529	23%	29 clinical/lab data	9 days	57	53%	Hierarchical ensemble classification	Hierarchical ensemble classification	RR, oxygen saturation, BUN, creatinine, anion gap, WBC, LDH, D‐dimer CRP, and ferritin
Douville et al. 2021 [24]	Cohort	United States	10‐fold cross validation	COVID‐19	398	23%	57 clinical/lab data	24 h	60 ± 17	53%	Random forest	Random forest	SpO2/FiO2 ratio, ventilatory frequency, and heart rate
Gorgojo‐Galindo et al. 2021 [25]	Cohort	Spain	Leave‐one‐out cross‐validation	COVID‐19	108	37%	29 clinical/lab data	During hospital stay	72.5 ± 15.25	55%	Logistic regression	Logistic regression	Glycemia, leukocytes, neutrophils, procalcitonin, CRP, ferritin, D‐dimer, and LDH
Jalali et al. 2021 [26]	Cohort	Iran	Leave‐one‐out cross‐validation	COVID‐19	137	16%	34 clinical/lab/imaging data	30 days	59.9 ± 16.8	62%	Logistic regression	Logistic regression	Ground glass nodule, patch B/punctate ground‐glass opacity, fibrous stripes, and air bronchogram sign with perihilar distribution, bilateral and ≥ 2 affected lobes
Kulkarni et al. 2021 [27]	Cohort	United States	10‐fold cross validation	COVID‐19	528	15%	Imaging data based on CXR	72 h	57.18	64.56%	DenseNet121	DenseNet121	—
Montomoli et al. 2021 [28]	Cohort	HENIVOT randomized clinical trial dataset	Internal validation	COVID‐19	109	40%	Clinical/lab data	During hospital stay	65	80%	XGBoost	XGBoost	Oxygen saturation, duration of the respiratory symptoms, the mean arterial pressure, and the P/F ratio
Shashikumar et al. 2021 [29]	Cohort	United States	10‐fold cross validation	COVID‐19	22416	5%	22 clinical/lab data	24 h	61.2	59%	VentNet (neural network)	VentNet (neural network)	Heart rate, oxygen saturation, RR, FIO2, and pH
Ebrahimian et al. 2021 [30]	Cohort	United States	Internal validation	COVID‐19	405	30%	Clinical/lab/imaging data	During hospital stay	65	65%	Neural network	Neural network	Total WBC counts, oxygen saturation < 93%
Yu et al. 2021 [31]	Cohort	United States	Internal validation	COVID‐19	1980	13%	34 clinical/lab data	During hospital stay	63.2	51.2%	XGBoost	XGBoost	Age, higher temperature, increased RR, and a lower oxygen saturation (SpO2)
Yamada et al. 2021 [32]	Cohort	Japan	External validation	COVID‐19	6873	21%	19 clinical/lab data	4 days	46.9 ± 20.1	58.80%	Logistic regression	Logistic regression	Sex, age, BMI, malignancy, fever, SOB, and wheezing
Afrash et al. 2022 [33]	Cohort	Iran	10‐fold cross‐validation	COVID‐19	482	45%	24 clinical/lab data	During hospital stay	—	—	XGBoost	XGBoost	—
Ayuso et al. 2022 [34]	Cohort	Spain	Fivefold cross‐validation	Influenza	992	2.20%	—	72 h	79	52.60%	Logistic regression	Logistic regression	Chronic obstructive pulmonary disease, immunosuppression, radiological abnormalities, RR, lymphopenia, lactate dehydrogenase, and C‐reactive protein at admission
Bardakci et al. 2022 [35]	Cohort	Turkey	Internal validation	COVID‐19	234	26%	18 clinical/lab/imaging data	During hospital stay	59	59.40%	Logistic regression	Logistic regression	Age, gender, D‐dimer, computed tomography severity score
Boussen et al. 2022 [36]	Cohort	France	Internal validation	COVID‐19	279	40%	10 clinical/lab data	48 h	63	67%	Gaussian mixture	Gaussian mixture	SpO2M, SpO2min, SpO2‐90, mean value of breathing frequency (BF)
Douglas et al. 2022 [37]	Cohort	United States	Fivefold cross‐validation	COVID‐19	3447	20%	—	48 h	49.4	48.50%	XGBoost	XGBoost	FiO2 (as fraction), Initial red blood cell count (million/uL), SpO2‐max (%), current lymphocyte count (thousand/uL), temperature (C), body weight (kg)
He et al. 2022 [38]	Cohort	United States	Fivefold cross‐validation	COVID‐19	50,703	12%	52 clinical/lab data	28 days	60.9	52.26%	XGBoost	XGBoost	Minimum SpO2 on admission, RR, lymphocyte count, BUN
Nopour et al. 2022 [39]	Cohort	Iran	10‐fold cross‐validation	COVID‐19	482	39%	15 clinical/lab data	During hospital stay	—	—	J‐48 decision tree, logistic regression, multilayer perception, Naïve Bayes	J‐48 decision tree	Dyspnea, cardiac diseases, pleural fluid, cough, and lymphocyte count
O′SHEA et al. 2022 [40]	Cohort	United States	10‐fold cross‐validation	COVID‐19	801	25.80%	20 clinical/lab/imaging data	7 days	59	58%	CNN	CNN	Cardiovascular disease, hypertension, and diabetes
Okuyucu et al. 2022 [41]	Cohort	Turkey	10‐fold cross‐validation	COVID‐19	733	14%	—	During hospital stay	—	—	Logistic regression	Logistic regression	C‐reactive protein, lactate dehydrogenase, neutrophil‐to‐lymphocyte ratio, age, lymphocyte count, and malignancy
Varzaneh et al. 2022 [42]	Cohort	Iran	10‐fold cross‐validation	COVID‐19	1225	13%	12 clinical/lab data	During hospital stay	—	—	Decision tree (DT), support vector nachine (SVM), multilayer perceptron (MLP), and K‐nearest neighbors(K‐NN)	Decision tree (DT)	High age, high weight, dry cough, fever, dyspnea, loss of smell, cardiovascular diseases, hypertension, C‐reactive protein, ALT/ASP, oxygen saturation (SPO2), and leukocytosis
Windham et al. 2022 [43]	Cohort	United States	10‐fold cross‐validation	COVID‐19	158	41%	28 clinical/lab/imaging data	24 h	56	51%	Logistic regression	Logistic regression	Diabetes mellitus, bilateral infiltrate on chest x‐ray, and elevated inflammatory markers
Xu et al. 2022 [44]	Cohort	Singapore	10‐fold cross‐validation	CAP	2302	14.90%	34 clinical/lab data	During hospital stay	—	—	Decision trees, random forests, XGBoost, support vector machines, Naïve Bayes, K‐nearest neighbors, ridge regression, logistic regression, and neural networks	XGBoost	Heart rate, SBP, glucose, DBP, cough, and BUN
Di Napoli et al. 2023 [45]	Cohort	Italy	Fivefold cross‐validation	COVID‐19	1051	12.20%	32 clinical/lab data	During hospital stay	69	59%	Neural networks	Neural networks	—
Lyu et al. 2023 [46]	Cohort	eICU‐CRD dataset	10‐fold cross‐validation	CAP	1676	17.70%	32 clinical/lab data	During hospital stay	69	54%	CatBoost, LightGBM, XGBoost, random forest	CatBoost	PT‐INR, age, sao2, respiration, and FiO2
Nopour et al. 2023 [47]	Cohort	Iran	Fivefold cross‐validation	COVID‐19	482	36.50%	18 clinical/lab data	During hospital stay	55.23 ± 6.66	52%	Neural networks	Neural networks	WBC count, BUN, activated PTT, absolute neutrophil count, and absolute lymphocyte count
Zhang et al. 2023 [48]	Cohort	United States	10‐fold cross‐validation	COVID‐19	73957	9%	40 clinical/lab data	During hospital stay	67	52.8%	Logistic regression	Logistic regression	Age, deficiency anemias, congestive heart failure, coagulopathy, diabetes with chronic complications, complicated hypertension, neurological disorders unaffecting movement, obesity, pulmonary circulation disease, severe renal failure, and weight loss
Lezcanoa et al. 2024 [49]	Cohort	Spain	Fivefold cross‐validation	COVID‐19	280	25.20%	34 clinical/lab data	During hospital stay	59	66%	Logistic regression	Logistic regression	Days from first symptoms to ICU admission, the APACHE II score, the oxygenation index, ROX index, blood procalcitonin, serum lactic dehydrogenase, total serum bilirubin, the dose of corticosteroids administered during the first 5 days of admission, lymphocyte count, pH, BMI, C‐reactive protein, time to oxygen therapy, and body temperature
Odeyemi et al. 2024 [50]	Cohort	United States	Fivefold cross‐validation	CAP	4379	10%	26 clinical/lab data	6 h	73.6	54%	Gradient boosting	Gradient boosting	RR, weight, BUN, systolic BP, age, bicarbonate, hematocrit, heart rate, and neutrophil count

### 2.7. Risk of Bias Assessment

Study quality was evaluated using the Prediction Model Risk of Bias Assessment Tool extended for Artificial Intelligence (PROBAST+AI), assessing participant selection, predictors, outcome definition, and analysis, with AI‐specific considerations like model transparency and fairness [51]. Each domain was rated as low, high, or unclear risk of bias, with an overall study rating. A “low risk” rating in PROBAST+AI indicates a relatively better methodological quality among ML studies, but still falls short of the methodological robustness typically expected in RCTs. Two reviewers independently conducted assessments, resolving disagreements through discussion or a third reviewer. Results were summarized in a risk of bias.

### 2.8. Certainty of Evidence

The certainty of evidence was assessed using the Grading of Recommendations Assessment, Development and Evaluation (GRADE) approach, adapted for prognostic studies [52]. Evidence started at a high‐quality rating and was downgraded for risk of bias (high PROBAST+AI ratings), inconsistency (I^2^ > 50%), indirectness (misaligned populations/outcomes), imprecision (wide confidence intervals), or publication bias (funnel plot asymmetry). Upgrading was considered for large effect sizes (e.g., AUROC > 0.9).

### 2.9. Data Synthesis

For meta‐analysis, true positive (TP), true negative (TN), false positive (FP), and false negative (FN) values were extracted directly from the included studies or derived from reported sensitivity and specificity within case/control groups. These values were then used to estimate pooled sensitivity and specificity using a bivariate random‐effects (HSROC) model implemented with the *midas* command in Stata (Version 18), which jointly models sensitivity and specificity. Outputs included pooled estimates with 95% confidence intervals, a summary receiver operating characteristic (SROC) curve to visualize sensitivity–specificity trade‐offs and a funnel plot with Deek′s test to assess publication bias. Heterogeneity was quantified using the I^2^ statistic (> 50% indicating substantial heterogeneity) and Cochran′s Q test (*p* < 0.10). Subgroup analyses examined differences by disease type (pneumonia, COVID‐19, and influenza), model type (deep learning vs. traditional ML), time frame, risk of bias, and country. To explore potential sources of heterogeneity, we conducted bivariate meta‐regression analyses using study‐level covariates with the *midas* command, jointly modeling sensitivity and specificity. For studies unsuitable for meta‐analysis due to inconsistent metrics, a narrative synthesis summarized study characteristics.

## 3. Results

### 3.1. Study Selection

**Figure 1:**
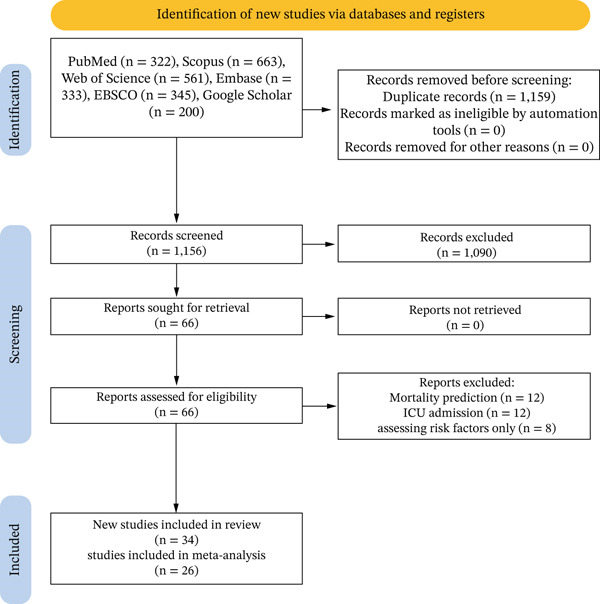
Study selection flow chart based on PRISMA 2020.

A comprehensive literature search identified 2315 records across major databases, including PubMed (*n* = 322), Scopus (*n* = 663), Web of Science (*n* = 561), Embase (*n* = 333), EBSCO (*n* = 345), and Google Scholar (*n* = 200). After cross‐database deduplication, 1159 duplicates were removed, leaving 1156 unique records for title and abstract screening. Of these, 1090 records were excluded as irrelevant. The remaining 66 reports were retrieved and assessed in full text. A total of 32 studies were excluded for the following reasons: mortality prediction (*n* = 12), ICU admission (*n* = 12), and assessment of risk factors only (*n* = 8). The systematic review ultimately included 34 studies, of which 26 provided sufficient performance data of ML models for quantitative synthesis (meta‐analysis) [17, 18, 20, 22–34, 36, 37, 39, 41, 44, 46–50]. The remaining eight studies were excluded from meta‐analysis due to an incomplete performance metrics reporting. The selection process was documented in a PRISMA flow diagram, with reasons for full‐text exclusions recorded. (Figure 1).

### 3.2. Baseline Characteristics

Among the included studies, only one study was a randomized controlled trial (RCT) [17], whereas the remaining were cohort studies. The total sample size across studies was 195,214 patients, with a mean age of 60.79 years (range: 47–79 years). Geographically, the studies were distributed as follows: 42% from the United States, 16% from Iran, 10% from Spain, and 32% from other countries. Validation methods varied, with 35% of studies using fivefold cross‐validation, 23% employing 10‐fold cross‐validation, 19% using a distinct 10‐fold cross‐validation approach, and 23% applying other methods. The number of features used in the models ranged from 10 to 90, with a mean of 29 features. Regarding pneumonia type, 87% of studies focused on COVID‐19, 10% on CAP, and 3% on influenza. Based on the best‐performing models reported, logistic regression was used in 29% of studies, XGBoost in 16%, random forest in 6%, and other models in 48%.

The studies identified a diverse set of features as most important for predicting intubation in pneumonia patients, reflecting the multifaceted nature of the condition. Clinical symptoms such as dyspnoea, respiratory rate, and wheezing were frequently highlighted, alongside vital signs including oxygen saturation, SpO2/FiO2 ratio, and heart rate. Laboratory markers emerged as significant, with C‐reactive protein, lactate dehydrogenase, D‐dimer, glycemia, white blood cell (WBC) count, and PT‐INR being commonly cited across studies. Radiological findings, including chest x‐ray severity and ground glass nodules, played a key role in several models. Comorbidities and patient characteristics, such as hypertension with complications, cardiac diseases, diabetes, presence of malignancy, and high age, were also critical predictors. Additionally, treatment‐related factors like the type of oxygen delivery used in the emergency department, FiO2 (as a fraction), along with the duration from first symptoms to ICU admission, were noted as influential features, underscoring the importance of both clinical and contextual data in these predictive models. Table 1 shows detailed characteristics of included studies.

### 3.3. Quality Assessment

**Figure 2:**
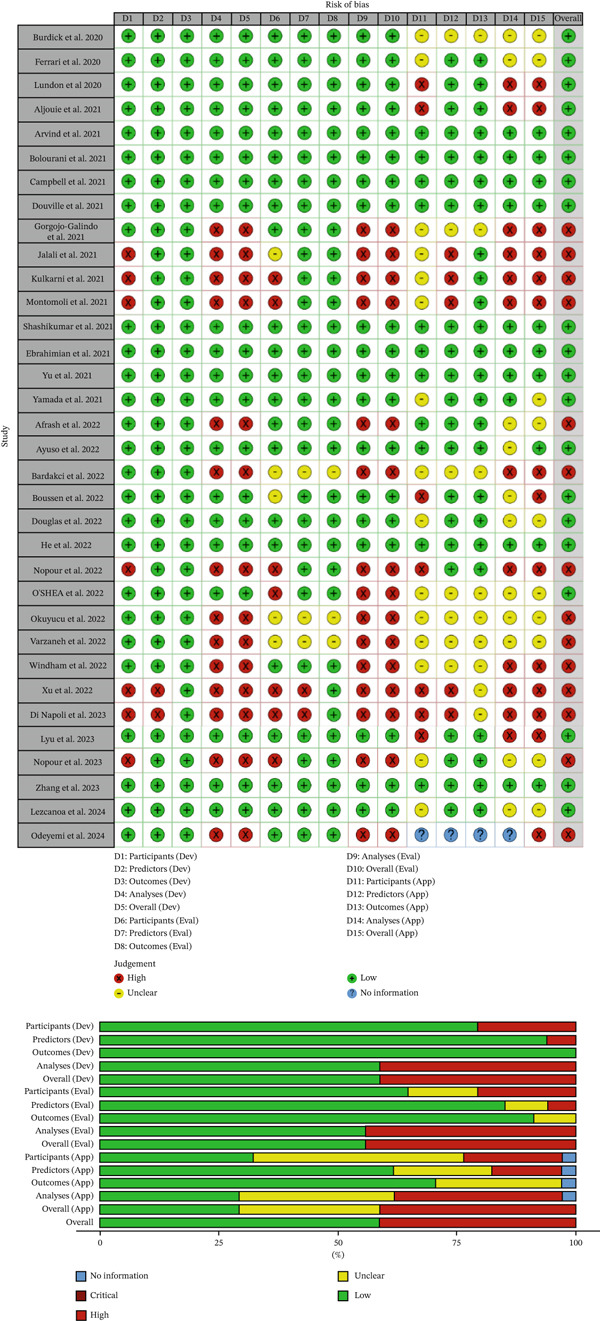
Quality assessment based on the PROBAST+AI tool.

The quality of the studies included in the meta‐analysis was evaluated using the PROBAST+AI tool, which assesses potential biases in studies involving AI models. The assessment focused on three key areas: development, evaluation, and application of the models. For the development phase, which examines how the models were built, 59% of the studies were found to have a low risk of bias, indicating that they followed robust methods, whereas 41% were rated as high risk, suggesting potential issues in their design or data handling. For the evaluation phase, which examines how the models′ performance was tested, 56% of studies were considered low risk, suggesting reliable testing methods. In comparison, 44% were high risk, indicating possible flaws in how the results were validated. For the application phase, which assesses how models are used in practical settings, 41% of studies were rated as high risk, meaning there were concerns about their real‐world reliability or clarity; 29% were unclear, indicating insufficient information to judge their quality; and 29% were low risk, showing good potential for practical use (Figure 2).

### 3.4. Overall Pooled Analysis

**Figure 3:**
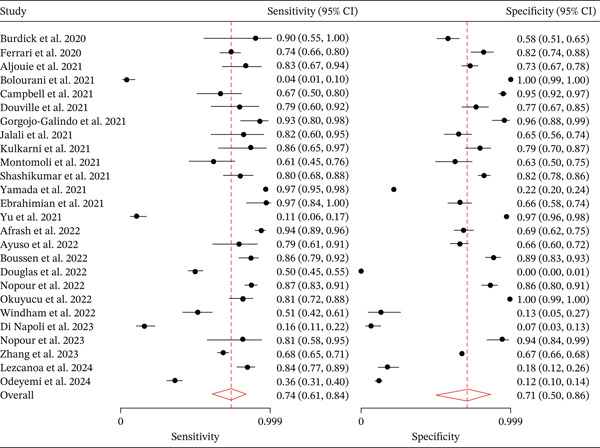
Forest plot for sensitivity and specificity based on performance metrics of best predicting algorithms.

**Figure 4:**
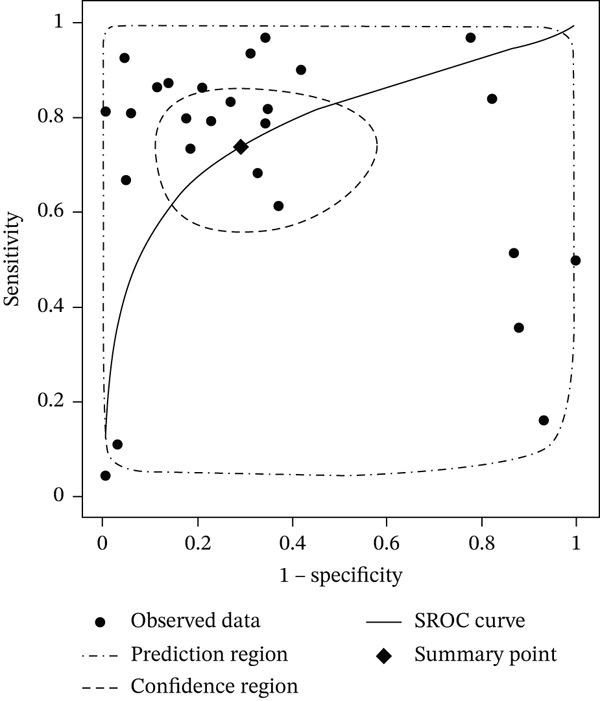
SROC curve specificity based on performance metrics of best predicting algorithms.

For the meta‐analysis, we included only the best‐performing model from each study as reported by the original authors. The pooled sensitivity was 0.74 (95% CI: 0.61–0.84) and the pooled specificity was 0.71 (95% CI: 0.50–0.86) (Figure 3). This indicates that, on average, ML approaches correctly identified approximately three‐quarters of TP cases while correctly ruling out about 70% of noncases. The summary AUROC was 0.79 (95% CI: 0.75–0.82) (Figure 4). In terms of secondary diagnostic measures, the pooled positive likelihood ratio was 2.5 (95% CI: 1.3–4.9) and the negative likelihood ratio was 0.37 (95% CI: 0.22–0.62). These likelihood ratios corresponded to a diagnostic odds ratio of 7 (95% CI: 2–20).

However, the analysis revealed significant between‐study variability. The I^2^ statistic was 90.45% for sensitivity and 94.58% for specificity and general I^2^ of 92%, pointing to substantial heterogeneity across studies. A detailed breakdown of performance metrics by model type is provided in Table 2.

**Table 2:** Performance metrics for different ML models. First line for each study indicates best performing model reported by original authors.

First author/year	Algorithm	Sensitivity	Specificity	AUROC (lower–upper CI)
Burdick et al. 2020 [17]	Logistic regression	0.9	0.58	0.866
Ferrari et al. 2020 [18]	LightGBM	0.74	—	0.84
Lundon et al. 2020 [19]	Decision tree (DT)	—	—	0.97 (1–0.94)
Aljouie et al. 2021 [20]	Support vector machines	0.84	0.73	0.87
Arvind et al. 2021 [21]	Random forest	—	—	0.84
Bolourani et al. 2021 [22]	XGBoost	0.051	0.994	0.77 (0.82–0.72)
	XGBoost+SMOTEENN	0.228	0.955	0.76 (0.72–0.8)
	Logistic regression	0.009	0.998	0.7 (0.65–0.75)
Campbell et al. 2021 [23]	Hierarchical ensemble classification	0.68	0.95	—
Douville et al. 2021 [24]	Random forest	0.8	0.77	—
Gorgojo‐Galindo et al. 2021 [25]	Logistic regression	0.917	0.95	0.94 (1–0.89)
Jalali et al. 2021 [26]	Logistic regression	0.797	0.655	0.755
Kulkarni et al. 2021 [27]	DenseNet121	0.8634	0.8438	—
Montomoli et al. 2021 [28]	XGBoost	0.61	0.63	—
Shashikumar et al. 2021 [29]	VentNet (neural network)	0.8	0.824	0.882 (0.892–0.878)
Ebrahimian et al. 2021 [30]	Neural networks	0.97	0.66	0.82
Yu et al. 2021 [31]	XGBoost	—	0.955	—
Yamada et al. 2021 [32]	Logistic regression	0.967	0.224	0.8
Afrash et al. 2022 [33]	XGBoost	0.907	0.765	0.853
Ayuso et al. 2022 [34]	Logistic regression	0.778	0.658	0.773 (0.859–0.687)
Bardakci et al. 2022 [35]	Logistic regression	—	—	0.914
Boussen et al. 2022 [36]	Gaussian mixture	—	—	0.94
Douglas et al. 2022 [37]	XGBoost	0.99	0.74	0.86
He et al. 2022 [38]	XGBoost	0.85	0.76	0.836 (0.84–0.833)
Nopour et al. 2022 [39]	J‐48 DT	0.869	—	0.892
	Logistic regression	0.701	—	—
	Multilayer perception	0.614	—	—
	Naïve Bayes	0.687	—	—
O′SHEA et al. 2022 [40]	CNN	—	—	0.82 (0.86–0.79)
Okuyucu et al. 2022 [41]	Logistic regression	0.8113	0.9952	—
Varzaneh et al. 2022 [42]	DT	0.927	0.934	—
	Support vector machines	0.692	0.832	—
	Multilayer perceptron	0.872	0.907	—
	K‐nearest neighbors	0.943	0.892	—
Windham et al. 2022 [43]	Logistic regression	0.91	0.41	0.83
Xu et al. 2022 [44]	XGBoost	0.108	—	0.801
	Ridge regression	0.039	—	0.711
	DTs	0.098	—	0.745
	Random forests	0.049	—	0.793
	K‐nearest neighbors	0.078	—	0.66
	Neural networks	0.049	—	0.694
	Support vector machines	0.049	—	0.759
	Naïve Bayes	0.961	—	0.707
	Logistic regression	0.137	—	0.686
Di Napoli et al. 2023 [45]	Neural Networks	0.747	0.457	0.64
Lyu et al. 2023 [46]	CatBoost	—	—	0.857
	LightGBM	—	—	0.831
	XGBoost	—	—	0.836
	Random forest	—	—	0.832
Nopour et al. 2023 [47]	Neural networks	0.898	0.951	0.906
Zhang et al. 2023 [48]	Logistic regression	0.681	0.674	0.715 (0.709–0.722)
Lezcanoa et al. 2024 [49]	Logistic regression	0.858	0.8	0.897 (0.875–0.862)
Odeyemi et al. 2024 [50]	Gradient boosting	0.72	0.57	0.713

### 3.5. Subgroup Analysis by Disease Type

Subgroup analysis of models applied to COVID‐19 patients (24 studies) resulted in an AUROC of 0.81 (95% CI: 0.77–0.84). Pooled sensitivity was 0.75 (95% CI: 0.61–0.85), and specificity was 0.74 (95% CI: 0.52–0.88), with a positive likelihood ratio of 2.8 (95% CI: 1.4–5.8), a negative likelihood ratio of 0.34 (95% CI: 0.20–0.57), and a diagnostic odds ratio of 8 (95% CI: 3–25). However, heterogeneity was considerable across studies (generalized I^2^ = 91.96*%*; sensitivity I^2^ = 89.97*%*; specificity I^2^ = 93.62*%*), indicating substantial variability in diagnostic performance estimates. Figure S1 shows a forest plot based on disease type.

### 3.6. Subgroup Analysis by Model Type

Subgroup analysis by algorithm type revealed distinct performance differences. Logistic regression models (nine studies) demonstrated an AUROC of 0.85 (95% CI: 0.81–0.88), with a sensitivity of 0.84 (95% CI: 0.72–0.91), specificity of 0.66 (95% CI: 0.32–0.89), a positive likelihood ratio of 2.5 (95% CI: 0.9–6.4), a negative likelihood ratio of 0.25 (95% CI: 0.11–0.53), and a diagnostic odds ratio of 10 (95% CI: 2–50). However, heterogeneity remained high, with generalized I^2^ = 89.91*%*, sensitivity I^2^ = 77.15*%*, and specificity I^2^ = 95.95*%*.

In contrast, XGBoost models (five studies) showed an AUROC of 0.52 (95% CI: 0.47–0.56), sensitivity of 0.40 (95% CI: 0.10–0.80), specificity of 0.72 (95% CI: 0.08–0.99), a positive likelihood ratio of 1.4 (95% CI: 0.2–11.0), a negative likelihood ratio of 0.84 (95% CI: 0.36–1.95), and a diagnostic odds ratio of 2 (95% CI: 0–29). Heterogeneity remained substantial in this subgroup, with generalized I^2^ = 78.42*%*, sensitivity I^2^ = 95.89*%*, and specificity I^2^ = 43.69*%*. Figure S2 shows the forest plot based on model type.

### 3.7. Subgroup Analysis by Country

Subgroup analysis by country highlighted geographic variability in model performance. Models from Iran (four studies) achieved an AUROC of 0.93 (95% CI: 0.90–0.95), with a sensitivity of 0.89 (95% CI: 0.84–0.93), specificity of 0.81 (95% CI: 0.66–0.90), a positive likelihood ratio of 4.7 (95% CI: 2.6–8.6), a negative likelihood ratio of 0.13 (95% CI: 0.09–0.18), and a diagnostic odds ratio of 36 (95% CI: 21–60). Heterogeneity in this subgroup was moderate, with generalized I^2^ = 0.13*%*, sensitivity I^2^ = 34.32*%*, and specificity I^2^ = 87.99*%*. Conversely, models from the United States (12 studies) had an AUROC of 0.67 (95% CI: 0.62–0.71), with a sensitivity of 0.61 (95% CI: 0.37–0.80), specificity of 0.67 (95% CI: 0.30–0.91), a positive likelihood ratio of 1.8 (95% CI: 0.7–5.0), a negative likelihood ratio of 0.59 (95% CI: 0.30–1.17), and a diagnostic odds ratio of 3 (95% CI: 1–16). Heterogeneity remained high in this subgroup, with generalized I^2^ = 91.82*%*, sensitivity I^2^ = 91.69*%*, and specificity I^2^ = 92.48*%*. These subgroup findings indicate significant variability in diagnostic accuracy across algorithms and regions, with persistent heterogeneity prompting further sensitivity analyses, as detailed subsequently. Figure S3 shows forest plot based on country.

### 3.8. Subgroup Analysis of Low Risk of Bias Studies

In the subgroup analysis restricted to studies rated as low risk of bias (PROBAST+AI), the pooled sensitivity was 0.73 (95% CI: 0.54–0.87), specificity was 0.71 (95% CI: 0.43–0.89), and AUROC was 0.78 (95% CI: 0.75–0.82). Substantial heterogeneity persisted across these studies (I^2^ = 90.4*%* for sensitivity and 96.4% for specificity and general I^2^ of 93%), indicating considerable variability even among methodologically robust investigations. The corresponding forest plot is presented in Figure S4.

### 3.9. Subgroup Analysis of Studies That Performed External Validation

Among studies that performed external validation (*n* = 6), the pooled diagnostic performance of ML models for predicting endotracheal intubation remained moderately high. The pooled sensitivity was 0.71 (95% CI: 0.31–0.93), specificity was 0.77 (95% CI: 0.32–0.96), and the summary AUROC was 0.80 (95% CI: 0.77–0.84). Substantial between‐study heterogeneity persisted (I^2^ = 90.1*%* for sensitivity and 92.9% for specificity), indicating considerable variation in performance estimates across externally validated models. The corresponding forest plot is presented in Figure S5.

### 3.10. Subgroup Analysis by Intubation Time

Subgroup analyses based on the prediction window for intubation revealed substantial variation in model performance. Models predicting intubation within 24 h (five studies) showed an AUROC of 0.73 (0.69–0.76), a pooled sensitivity of 0.65 (95% CI: 0.46–0.80) and specificity of 0.49 (95% CI: 0.22–0.77), with heterogeneity indicated by generalized I^2^ = 0.18*%*, sensitivity I^2^ = 79.97*%*, and specificity I^2^ = 96.92*%* (Figure S6). For 48‐h prediction windows (four studies), the pooled AUROC was 0.57 (95% CI: 0.53–0.62), sensitivity 0.49 (95% CI: 0.13–0.86), and specificity 0.70 (95% CI: 0.04–0.99), with generalized I^2^ = 76.42*%*, sensitivity I^2^ = 97.50*%*, and specificity I^2^ = 23.66*%* (Figure S7). Models predicting intubation within 72 h (four studies) achieved an AUROC of 0.83 (95% CI: 0.80–0.86), sensitivity 0.64 (95% CI: 0.27–0.90), and specificity 0.83 (95% CI: 0.62–0.94); heterogeneity remained notable (generalized I^2^ = 0.11*%*, sensitivity I^2^ = 89.75*%*, and specificity I^2^ = 95.67*%*) (Figure S8). The broadest time frame intubation during hospitalization (13 studies) showed an AUROC of 0.86 (95% CI: 0.83–0.89), pooled sensitivity 0.82 (95% CI: 0.69–0.91), and specificity 0.74 (95% CI: 0.48–0.90), though heterogeneity persisted (generalized I^2^ = 91.96*%*, sensitivity I^2^ = 87.65*%*, and specificity I^2^ = 95.09*%*) (Figure S9).

### 3.11. The Leave‐One‐Out Sensitivity Analysis

The leave‐one‐out sensitivity analysis demonstrated that the overall pooled effect size was robust and not disproportionately influenced by any single study. When each study was sequentially omitted, the effect size estimates remained consistent, ranging from 1.76 to 2.19, with all confidence intervals overlapping the main pooled estimate (1.90, 95% CI: 0.82–2.98). The statistical significance was maintained across all iterations (*p* ≤ 0.001), indicating the stability of the findings. Notably, the exclusion of studies such as Douglas et al. (2022) [37], Windham et al. (2022) [43], Di Napoli et al. (2023) [45], and Odeyemi et al. (2024) [50] produced slightly higher effect sizes, but these changes did not materially alter the overall interpretation. Collectively, these results confirm that the meta‐analysis findings are reliable and not driven by any individual study (Figure S10) (Table S3).

### 3.12. Meta‐Regression Analysis

Meta‐regression was conducted to evaluate whether pneumonia type, model type, feature composition, study country, overall risk of bias, study period, validation strategy, or sample size contributed to between‐study heterogeneity. None of these covariates significantly accounted for variability across models (all *p* > 0.05), and heterogeneity remained unexplained (I^2^ = 0*%*). These findings indicate that unmeasured factors such as dataset composition, preprocessing pipelines, or reporting inconsistency may underlie the observed variation in model performance. (Table S4).

### 3.13. Publication Bias

**Figure 5:**
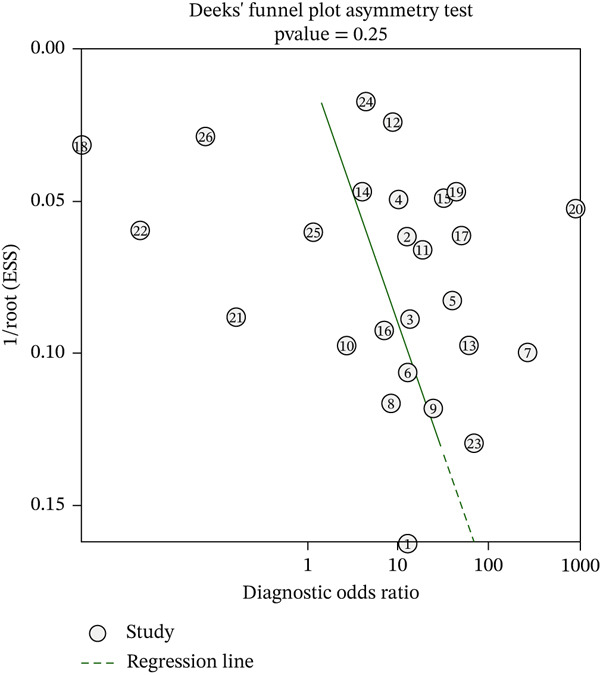
Funnel plot assessing publication bias. Visual asymmetry suggests possible selective reporting despite a nonsignificant Deeks′ test (*p* = 0.252), indicating potential overestimation of pooled AUROC.

Despite nonsignificant results from formal publication bias tests (Deeks′ test *p* value = 0.252), the funnel plot (Figure 5) displayed notable asymmetry, with an absence of small studies showing low AUROCs (< 0.70). This likely reflects selective publication of positive results, a known issue in ML research, suggesting that the pooled AUROC of 0.79 may overstate the true model performance, which could be closer to the lower bound of the confidence interval (0.75) or even lower.

### 3.14. Certainty of Evidence

The certainty of evidence was appraised using the GRADE framework for diagnostic test accuracy studies. Evidence certainty was downgraded mainly due to substantial heterogeneity across all subgroups (high I^2^ values). For the overall meta‐analysis, the certainty was rated as moderate, as consistent diagnostic accuracy was observed despite between‐study variability. Subgroups analyzing COVID‐19 cohorts and logistic regression models also showed moderate certainty due to heterogeneity and variable specificity. In contrast, subgroups for XGBoost models and U.S.‐based studies were rated low because of lower diagnostic accuracy, wide confidence intervals, and residual heterogeneity. Although publication bias was not statistically significant, a notable asymmetry in the funnel plot and the proportion of studies with high or unclear PROBAST+AI risk further limited confidence in the evidence. (Table S5).

## 4. Discussion

The systematic review of 34 studies (26 studies in meta‐analysis), involving 195,214 pneumonia patients, reveals that ML algorithms provide modest accuracy for predicting intubation. We focused on AUROC as the primary measure of model performance, as it was consistently reported and provides a prevalence‐independent assessment of discriminative ability. The pooled AUROC of 0.79 indicates modest performance, suggesting that current ML models may support but not replace clinical judgment for intubation decisions as significant heterogeneity was observed across studies. This marked heterogeneity across studies (I^2^ > 90*%*) likely stems from the high risk of bias identified by the PROBAST+AI tool. Frequent reliance on internal validation, absence of prespecified modeling protocols, and unclear handling of missing data and feature selection undermine the reliability and generalizability of most reported performance metrics.

Logistic regression models accuracy (AUROC 0.85, 95% CI: 0.81–0.88) in predicting intubation risk, suggesting potential clinical utility for early intervention and monitoring. However, this apparent superiority likely reflects a high risk of bias and possible publication bias rather than true algorithmic advantage. Conversely, the poor performance of XGBoost models may stem from suboptimal tuning, small sample sizes, or overfitting. The observed Iran–USA discrepancy likely reflects differences in data quality, patient populations, and reporting standards rather than true algorithmic performance, underscoring that reporting variability is the key driver of outcome differences.

Several studies demonstrated robust design and clinically relevant outcomes. Shashikumar et al. developed VentNet, achieving AUROC = 0.886 for predicting mechanical ventilation within 24 h and 0.943 in external COVID‐19 cohorts [29], with respiratory rate emerging as a key predictor. Burdick et al. reported XGBoost outperforming MEWS (AUROC = 0.866; sensitivity = 0.90) using only early admission data, supporting rapid clinical triage [17].

Multimodal models further improved accuracy. Di Napoli et al. integrated 3D‐ResNet chest CT with clinical features (91.3% accuracy) [45], whereas Ebrahimian et al. showed that combining AI‐derived chest x‐ray scores (AUROC = 0.82) with clinical data outperformed the RALE score (0.87), reaching 0.90 overall [30]. This suggests a practical advantage in resource‐constrained environments, where speed and efficiency are critical.

Interpretability was addressed by Lezcanoa et al. using GLMM‐trees (AUROC = 0.87) that identified APACHE II and ROX index as key features [49], and by Douville et al., whose random forest achieved C − statistic = 0.885 and highlighted SpO₂/FiO₂ ratio as a key longitudinal marker [24]. These findings emphasize the importance of continuous monitoring in predicting deterioration.

Patient‐specific studies reinforced clinical applicability. Odeyemi et al. reported a gradient boosting machine for CAP (AUROC = 0.713) identifying respiratory rate and blood urea nitrogen as major predictors, outperforming PSI and CURB‐65 [50]. Ferrari et al. used LightGBM to show biomarker‐based models surpassed symptom‐based ones [18], suggesting laboratory‐driven feature sets may reduce FNs and guide data standardization in real‐world implementation.

Earlier predictive efforts, particularly during the COVID‐19 era, relied primarily on clinical, immunological, and radiographic indicators. Detsika et al. identified immune markers such as the CD8^+^:B‐cell ratio (AUROC = 0.795) and neutrophil‐to‐lymphocyte ratio (AUROC = 0.783) as strong individual predictors, with combined metrics achieving AUROC = 0.845 (sensitivity = 77.9*%*, specificity = 85.1*%*) [53]. Similarly, Jaramillo et al. reported that respiratory indices—including the right U.D.E.S.I. index (AUROC = 0.798, specificity = 82.4*%*) and the ROX index—outperformed traditional oxygenation measures for early detection of mechanical ventilation needs [54]. However, such conventional approaches remain limited by dependence on fixed thresholds, manual data extraction, and linear modeling assumptions, which reduce reproducibility and scalability. For instance, Detsika et al. found weaker discrimination for the CD4^+^:CD8^+^ ratio (AUROC = 0.664) [53], whereas Jaramillo et al. reported missing physiologic data compromising model completeness [54]. As noted by Xu et al., multicollinearity in classical models can obscure nonlinear associations [55], underscoring the rationale for adopting ML frameworks capable of integrating complex, multimodal predictors.

In contrast to traditional approaches, ML models demonstrate superior predictive capacity by integrating diverse data sources—including clinical, laboratory, and imaging variables—into unified, adaptive frameworks. Their ability to model nonlinear interactions and accommodate complex interdependencies, as shown by Xu et al. in COVID‐19 risk stratification, enables more accurate and individualized predictions of intubation needs [55]. Unlike conventional models that rely on fixed biomarker thresholds or imaging scores and thus exhibit limited scalability, ML systems dynamically adapt to heterogeneous populations. For instance, Torres‐Vargas et al. reported a modest AUROC of 0.61 for the RALE score [56], whereas predictive scores from Anderson et al. and Bello‐Chavolla et al. achieved C‐statistics near 0.82 but lacked robustness across patient subgroups influenced by comorbidities such as obesity and diabetes [57, 58]. Collectively, this adaptability marks a substantial advancement toward real‐time, context‐aware clinical decision support compared with traditional, static predictive methods.

## 5. Bias and Reliability

According to PROBAST frameworks, high‐risk bias frequently originates from methodological weaknesses across the domains of data selection, measurement, analysis, and validation. Selection bias is especially pervasive: datasets predominantly comprise hospitalized or critically ill cohorts from large urban tertiary centers, leading to overrepresentation of severe pneumonia cases and underrepresentation of outpatient or rural populations [27, 39]. As a result, model performance is often inflated in internal validation but fails to generalize across healthcare settings with differing case mixes [28]. The problem is compounded by demographic and socioeconomic imbalances, with older adults, women, and minority groups systematically underrepresented, contributing to poor calibration and unequal predictive performance across subgroups [45, 47].

Measurement and label bias also pose critical threats to validity. The decision to initiate ventilation is not a purely physiological endpoint but one deeply influenced by institutional protocols, staffing ratios, and evolving clinical thresholds, which vary substantially across time and geography [44]. For instance, during pandemic surges, thresholds for intubation shifted in response to ventilator scarcity, embedding nonclinical variability into outcome labels [57, 58]. Many studies further depend on proxy indicators such as ICD codes or oxygen supplementation levels, which may misclassify disease severity and blur the distinction between true ventilatory need and clinical discretion [36, 46]. The frequent class imbalance, where only a small fraction of pneumonia patients require mechanical ventilation, reinforces model bias by favoring nonintubation predictions and yielding deceptively high accuracy despite poor sensitivity for critical cases [29, 34].

Algorithmic and feature selection biases add further complexity. When input variables are not standardized or are heavily correlated, models like random forests or deep neural networks may amplify spurious associations, particularly in small samples [44, 48]. Moreover, extensive hyperparameter tuning and repeated optimization cycles on limited datasets frequently result in overfitting that inflates AUC metrics but collapses under external validation.

Temporal and confounding biases also remain underappreciated. Concept drift, where model performance degrades due to shifts in disease presentation, treatment protocols, or population immunity, is particularly relevant in COVID‐19 pneumonia. Models trained on early pandemic data, dominated by unvaccinated, high‐severity cases, often overestimate ventilation needs in later, vaccinated cohorts [17, 18, 58]. Even comparative analyses intended to benchmark model performance introduce secondary layers of bias.

Inadequate stratification by pneumonia type, region, or ethnicity obscures heterogeneity and prevents fair comparison across studies. Furthermore, publication and replication biases persist: studies showing no improvement of ML over conventional scores are less likely to be published, and code opacity limits reproducibility.

## 6. Clinical Implication and Challenges

ML models for predicting the need for intubation in pneumonia patients hold considerable promise for improving clinical decision‐making, particularly in acute and intensive care settings where early identification of respiratory failure is critical. However, translating these algorithms from research to bedside practice requires rigorous methodological standards to ensure reliability, generalizability, and patient safety. A key priority is the standardization of data collection and outcome definitions across studies. Current literature exhibits wide variability in defining intubation criteria, timing, and clinical thresholds for respiratory deterioration, which undermines comparability and inflates performance estimates. Consistent use of standardized clinical parameters, such as oxygenation indices, vital sign trajectories, or established severity scores, would enhance model robustness and facilitate integration into diverse healthcare environments. Similarly, harmonized preprocessing of EHR data is essential to reduce institutional bias arising from irregular measurements or missing variables. Equally important is rigorous external validation. Most available models have been developed in single‐center retrospective cohorts with high or unclear risks of bias, limiting their generalizability to broader populations. Validation across multiple institutions and demographic groups, supported by transparent reporting frameworks like TRIPOD, will be crucial to confirm model stability and real‐world applicability [59]. Finally, prospective trials are indispensable to determine whether algorithm‐guided interventions truly improve outcomes, such as reducing delayed intubations, optimizing resource use, or lowering mortality. These studies should also address explainability and workflow integration to ensure clinician trust and practical adoption. Until such evidence emerges, implementation should remain cautious, emphasizing transparency and external validation over premature deployment.

## 7. Limitations

The review faced challenges due to the predominance of cohort studies, which may introduce selection bias compared with RCTs. Variability in validation methods and the number of features used across studies could affect the comparability of results. Another limitation is the lack of consistent reporting on model interpretability. Only a few studies included explainability analyses using SHAP or comparable techniques. This absence of transparency limits insight into how predictive features contribute to intubation risk and may hinder clinical adoption. The focus on specific pneumonia types, particularly COVID‐19, might limit the applicability to other respiratory conditions. Unexplored factors, such as detailed patient demographics or institutional practices, may also contribute to the observed variability, highlighting areas for further investigation.

## 8. Conclusion

This systematic review indicates that, although ML algorithms show modest overall performance in predicting intubation in pneumonia, the current evidence is limited by substantial heterogeneity, a high risk of bias, and inconsistent development and validation practices. Existing ML models lack the reliability and standardization required for safe clinical use. To enable reliable clinical implementation, future research should prioritize transparent, prospective, multicenter validation studies that use standardized data collection protocols and clinically feasible feature sets.

**Nomenclature**
LR: logistic regression
LGBM: LightGBM
SVM: support vector machine
RF: random forest
XGB: XGBoost
XGB+SMOTEENN: XGBoost with SMOTEENN (Synthetic Minority Over‐sampling Technique Edited Nearest Neighbor)
HEC: Hierarchical ensemble classification
DN121: DenseNet121
VentNet: ventilator prediction neural network
NN: neural network
GM: Gaussian mixture
J48: J‐48 decision tree
MLP: multilayer perceptron
NB: Naïve Bayes
GB: gradient boosting

## Author Contributions

E.A. was a main contributor in the design and implementation. P.E. and E.A. independently assessed articles and extracted data independently. P.E. and E.A. were responsible for drafting the manuscript. S.F. and M.F. performed statistical analysis.

## Funding

No funding was received for this manuscript.

## Disclosure

All authors read and approved the final manuscript.

## Ethics Statement

The authors have nothing to report.

## Consent

The authors have nothing to report.

## Conflicts of Interest

The authors declare no conflicts of interest.

## Supporting information


**Supporting Information** Additional supporting information can be found online in the Supporting Information section. PRISMA 2020 Checklist. Table S1: PICO framework. Table S2: Search syntax for different databases. Table S3: The leave‐one‐out sensitivity analysis. Table S4: Meta‐regression results. Table S5: Summary of findings for sensitivity and specificity with explicit. GRADE downgrades by domain. Figure S1: Forest plot based on disease type. Figure S2: Forest plot based on model type. Figure S3: Forest plot based on country. Figure S4: Forest plot based on low–risk of bias studies. Figure S5: Forest plot based on studies that performed external validation. Figure S6: Forest plot based on studies predicted intubation within 24 h. Figure S7: Forest plot based on studies predicted intubation within 48 h. Figure S8: Forest plot based on studies predicted intubation within 72 h. Figure S9: Forest plot based on studies predicted intubation during hospitalization period. Figure S10: The leave‐one‐out sensitivity analysis.

## Data Availability

All data generated or analyzed during this study are included in this published article (and its supporting information files).
