# Supplementary material for: Optimizing Intubation Prediction in Pneumonia Patients: A Systematic Review and Meta‐Analysis of Machine Learning Algorithms
Source: Pulm Med. 2026 Mar 18;2026:6670267. doi: 10.1155/pm/6670267 (PMC13140312; doi:10.1155/pm/6670267)
Supplement: Supplementary file 1 — Supporting Information Additional supporting information can be found online in the Supporting Information section. PRISMA 2020 Checklist. Table S1: PICO framework. Table S2: Search syntax for different databases. Table S3: The leave‐one‐out sensitivity analysis. Table S4: Meta‐regression results. Table S5: Summary of findings for sensitivity and specificity with explicit. GRADE downgrades by domain. Figure S1: Forest plot based on disease type. Figure S2: Forest plot based on model type. Figure S3: Forest plot based on country. Figure S4: Forest plot based on low–risk of bias studies. Figure S5: Forest plot based on studies that performed external validation. Figure S6: Forest plot based on studies predicted intubation within 24 h. Figure S7: Forest plot based on studies predicted intubation within 48 h. Figure S8: Forest plot based on studies predicted intubation within 72 h. Figure S9: Forest plot based on studies predicted intubation during hospitalization period. Figure S10: The leave‐one‐out sensitivity analysis. [file PM-2026-6670267-s001.docx]

| **PRISMA 2020 Checklist** | | | |
| --- | --- | --- | --- |
| **Section and Topic** | **Item #** | **Checklist Item** | **Location in Manuscript** |
| **TITLE** | 1 | Identify the report as a systematic review. | Title page |
| **ABSTRACT** | 2 | See the PRISMA 2020 for Abstracts checklist. | Page 1 |
| **INTRODUCTION** | 3 | Describe the rationale for the review in the context of existing knowledge. | Pages 2–3 |
|  | 4 | Provide an explicit statement of the objective(s) or question(s) the review addresses. | Page 3 |
| **METHODS** | 5 | Specify the inclusion and exclusion criteria for the review and how studies were grouped for the syntheses. | Pages 4–5 |
|  | 6 | Specify all databases, registers, websites, organisations, reference lists and other sources searched or consulted to identify studies. Specify the date when each source was last searched or consulted. | Page 4 |
|  | 7 | Present the full search strategies for all databases, registers and websites, including any filters and limits used. | Page 4; Supplementary Table 2 |
|  | 8 | Specify the methods used to decide whether a study met the inclusion criteria of the review, including how many reviewers screened each record and each report retrieved, whether they worked independently, and if applicable, details of automation tools used in the process. | Page 5 |
|  | 9 | Specify the methods used to collect data from reports, including how many reviewers collected data from each report, whether they worked independently, any processes for obtaining or confirming data from study investigators, and if applicable, details of automation tools used in the process. | Page 5 |
|  | 10a | List and define all outcomes for which data were sought. Specify whether all results that were compatible with each outcome domain in each study were sought (e.g. for all measures, time points, analyses), and if not, the methods used to decide which results to collect. | Pages 5–6 |
|  | 10b | List and define all other variables for which data were sought (e.g. participant and intervention characteristics, funding sources). Describe any assumptions made about any missing or unclear information. | Pages 5–6 |
|  | 11 | Specify the methods used to assess risk of bias in the included studies, including details of the tool(s) used, how many reviewers assessed each study and whether they worked independently, and if applicable, details of automation tools used in the process. | Page 6 (PROBAST+AI) |
|  | 12 | Specify for each outcome the effect measure(s) (e.g. risk ratio, mean difference) used in the synthesis or presentation of results. | Page 7 |
|  | 13a | Describe the processes used to decide which studies were eligible for each synthesis (e.g. tabulating study characteristics and comparing them against the planned groups for each synthesis). | Page 7 |
|  | 13b | Describe any methods required to prepare the data for presentation or synthesis, such as handling of missing summary statistics or data conversions. | Page 7 |
|  | 13c | Describe any methods used to tabulate or visually display results of individual studies and syntheses. | Tables 1–2; Figures 3–5 |
|  | 13d | Describe any methods used to synthesize results and provide a rationale for the choice(s). If meta-analysis was performed, describe the model(s), methods to identify heterogeneity, and software package(s) used. | Page 7 (HSROC, bivariate random-effects using MIDAS in Stata 18) |
|  | 13e | Describe any methods used to explore possible causes of heterogeneity among study results (e.g. subgroup analysis, meta-regression). | Page 7; Supplementary Table 4 |
|  | 13f | Describe any sensitivity analyses conducted to assess robustness of the synthesized results. | Page 8; Supplementary Figure 10 |
|  | 14 | Describe any methods used to assess risk of bias due to missing results in a synthesis (arising from reporting biases). | Page 8 (Deeks’ test, funnel plot) |
|  | 15 | Describe any methods used to assess certainty (or confidence) in the body of evidence for an outcome. | Page 8 (GRADE assessment) |
| **RESULTS** | 16a | Describe the results of the search and selection process, from the number of records identified in the search to the number of studies included in the review, ideally using a flow diagram. | Page 8; Figure 1 |
|  | 16b | Cite studies that might appear to meet the inclusion criteria but were excluded, and explain why they were excluded. | Page 8 |
|  | 17 | Cite each included study and present its characteristics. | Pages 9–10; Table 1 |
|  | 18 | Present assessments of risk of bias for each included study. | Page 10; Figure 2 |
|  | 19 | For all outcomes, present, for each study, summary statistics for each group and an effect estimate with its precision (e.g. confidence interval), ideally using structured tables or plots. | Pages 10–12; Table 2 |
|  | 20a | For each synthesis, briefly summarize the characteristics and risk of bias among contributing studies. | Page 12 |
|  | 20b | Present results of all statistical syntheses conducted, including summary estimates and measures of statistical heterogeneity. | Pages 12–13; Figures 3–4 |
|  | 20c | Present results of all investigations of possible causes of heterogeneity among study results. | Page 8; Supplementary Table 4 |
|  | 20d | Present results of all sensitivity analyses conducted to assess the robustness of the synthesized results. | Page 8; Supplementary Figure 10 |
|  | 21 | Present assessments of risk of bias due to missing results (arising from reporting biases) for each synthesis assessed. | Page 8; Figure 5 |
|  | 22 | Present assessments of certainty (or confidence) in the body of evidence for each outcome assessed. | Page 8–9; Supplementary Table 5 |
| **DISCUSSION** | 23a | Provide a general interpretation of the results in the context of other evidence. | Pages 13–15 |
|  | 23b | Discuss any limitations of the evidence included in the review. | Page 15 |
|  | 23c | Discuss any limitations of the review processes used. | Page 15 |
|  | 23d | Discuss implications of the results for practice, policy, and future research. | Pages 15–16 |
| **OTHER INFORMATION** | 24a | Provide registration information for the review, including register name and registration number, or state that the review was not registered. | Page 4 (PROSPERO CRD420251064236) |
|  | 24b | Indicate where the review protocol can be accessed, or state that a protocol was not prepared. | Page 4 |
|  | 24c | Describe and explain any amendments to information provided at registration or in the protocol. | Page 4 |
|  | 25 | Describe sources of financial or non-financial support for the review and the role of the funders or sponsors in the review. | Page 16 (“Funding: None”) |
|  | 26 | Declare any competing interests of review authors. | Page 16 (“No competing interests”) |
|  | 27 | Report which of the following are publicly available and where they can be found: data collection forms, extracted data, analysis datasets, code, or other materials. | Page 16 (“All data included in this article and supplementary files”) |

| Supplementary table 1. PICO Framework. | |
| --- | --- |
| Population (P) | Adult patients (aged ≥18 years) diagnosed with pneumonia (community-acquired, hospital-acquired, or ventilator-associated), COVID-19, or influenza, admitted to hospital settings, including emergency departments, wards, or intensive care units. |
| Intervention (I) | Use of machine learning algorithms, including artificial intelligence and deep learning models such as Support Vector Machines, Decision Trees, Logistic Regression, Random Forest, K-Nearest Neighbors, and Artificial Neural Networks, to predict intratracheal intubation, endotracheal intubation, respiratory insufficiency, respiratory failure, respiratory depression, or ventilatory depression. |
| Comparator (C) | Not explicitly defined as a specific comparator was not required; however, comparisons were made implicitly against traditional clinical prediction tools or no intervention where applicable, depending on the study design. |
| Outcome (O) | Primary outcomes included performance metrics such as Area Under the Receiver Operating Characteristic Curve (AUC), sensitivity, specificity, positive predictive value (PPV), negative predictive value (NPV), and accuracy. Secondary outcomes encompassed key predictors, model type performance, and clinical utility. |

| Supplementary table 2. Search syntax for different databases. | | |
| --- | --- | --- |
| Database | Search syntax | results |
| Pubmed | ("Machine Learning"[MeSH Terms] OR "Machine Learning"[tiab] OR "Artificial Intelligence"[MeSH Terms] OR "Artificial Intelligence"[tiab] OR "Deep Learning"[tiab] OR "prediction model*"[tiab] OR "predictive model*"[tiab]) AND ("Intubation, Intratracheal"[MeSH Terms] OR "Intratracheal Intubation*"[tiab] OR "Intubation"[tiab] OR "Endotracheal Intubation*"[tiab] OR "Respiratory Insufficiency"[MeSH Terms] OR "Respiratory Insufficiency"[tiab] OR "Respiratory Failure*"[tiab] OR "Respiratory Depression"[tiab] OR "Ventilatory Depression"[tiab]) AND ("Pneumonia"[MeSH Terms] OR "Pneumonia*"[tiab] OR "Lung Inflammation*"[tiab] OR "Pneumonitis"[MeSH Terms] OR "Pneumonitis"[tiab] OR "Pneumonitides"[tiab] OR "Pulmonary Inflammation*"[tiab] OR "COVID-19"[MeSH Terms] OR "COVID-19"[tiab] OR "COVID 19"[tiab] OR "SARS Coronavirus 2 Infection*"[tiab] OR "2019-nCoV"[tiab] OR "2019 nCoV"[tiab] OR "SARS-CoV-2"[tiab] OR "SARS CoV 2"[tiab] OR "Coronavirus"[MeSH Terms] OR "Coronavirus"[tiab] OR "COVID19"[tiab] OR "Influenza, Human"[MeSH Terms] OR "Influenza*"[tiab] OR "Grippe"[tiab] OR "Flu"[tiab]) | 322 |
| Scopus | TITLE-ABS-KEY(("Machine Learning" OR "Artificial Intelligence" OR "Deep Learning" OR "prediction model*" OR "predictive model*") AND ("Intratracheal Intubation*" OR "Intubation" OR "Endotracheal Intubation*" OR "Respiratory Insufficiency" OR "Respiratory Failure*" OR "Respiratory Depression" OR "Ventilatory Depression") AND ("Pneumonia*" OR "Lung Inflammation*" OR "Pneumonitis" OR "Pneumonitides" OR "Pulmonary Inflammation*" OR "COVID-19" OR "COVID 19" OR "SARS Coronavirus 2 Infection*" OR "2019-nCoV" OR "2019 nCoV" OR "SARS-CoV-2" OR "SARS CoV 2" OR "Coronavirus" OR "COVID19" OR "Influenza*" OR "Grippe" OR "Flu")) | 663 |
| Web of science | (TS=("Machine Learning" OR "Artificial Intelligence" OR "Deep Learning" OR "prediction model*" OR "predictive model*") OR AB=("Machine Learning" OR "Artificial Intelligence" OR "Deep Learning" OR "prediction model*" OR "predictive model*") OR AK=("Machine Learning" OR "Artificial Intelligence" OR "Deep Learning" OR "prediction model*" OR "predictive model*")) AND (TI=("Intratracheal Intubation*" OR "Intubation" OR "Endotracheal Intubation*" OR "Respiratory Insufficiency" OR "Respiratory Failure*" OR "Respiratory Depression" OR "Ventilatory Depression") OR AB=("Intratracheal Intubation*" OR "Intubation" OR "Endotracheal Intubation*" OR "Respiratory Insufficiency" OR "Respiratory Failure*" OR "Respiratory Depression" OR "Ventilatory Depression") OR AK=("Intratracheal Intubation*" OR "Intubation" OR "Endotracheal Intubation*" OR "Respiratory Insufficiency" OR "Respiratory Failure*" OR "Respiratory Depression" OR "Ventilatory Depression")) AND (TI=("Pneumonia*" OR "Lung Inflammation*" OR "Pneumonitis" OR "Pneumonitides" OR "Pulmonary Inflammation*" OR "COVID-19" OR "COVID 19" OR "SARS Coronavirus 2 Infection*" OR "2019-nCoV" OR "2019 nCoV" OR "SARS-CoV-2" OR "SARS CoV 2" OR "Coronavirus" OR "COVID19" OR "Influenza*" OR "Grippe" OR "Flu") OR AB=("Pneumonia*" OR "Lung Inflammation*" OR "Pneumonitis" OR "Pneumonitides" OR "Pulmonary Inflammation*" OR "COVID-19" OR "COVID 19" OR "SARS Coronavirus 2 Infection*" OR "2019-nCoV" OR "2019 nCoV" OR "SARS-CoV-2" OR "SARS CoV 2" OR "Coronavirus" OR "COVID19" OR "Influenza*" OR "Grippe" OR "Flu") OR AK=("Pneumonia*" OR "Lung Inflammation*" OR "Pneumonitis" OR "Pneumonitides" OR "Pulmonary Inflammation*" OR "COVID-19" OR "COVID 19" OR "SARS Coronavirus 2 Infection*" OR "2019-nCoV" OR "2019 nCoV" OR "SARS-CoV-2" OR "SARS CoV 2" OR "Coronavirus" OR "COVID19" OR "Influenza*" OR "Grippe" OR "Flu")) | 561 |
| Embase | (('machine learning'/exp OR 'artificial intelligence'/exp OR 'deep learning'/exp OR "Machine Learning":ti,ab,kw OR "Artificial Intelligence":ti,ab,kw OR "Deep Learning":ti,ab,kw OR "prediction model*":ti,ab,kw OR "predictive model*":ti,ab,kw) AND ('respiratory tract intubation'/exp OR 'respiratory failure'/exp OR "Intratracheal Intubation*":ti,ab,kw OR "Intubation":ti,ab,kw OR "Endotracheal Intubation*":ti,ab,kw OR "Respiratory Insufficiency":ti,ab,kw OR "Respiratory Failure*":ti,ab,kw OR "Respiratory Depression":ti,ab,kw OR "Ventilatory Depression":ti,ab,kw) AND ('pneumonia'/exp OR 'coronavirus disease 2019'/exp OR "Pneumonia*":ti,ab,kw OR "Lung Inflammation*":ti,ab,kw OR "Pneumonitis":ti,ab,kw OR "Pneumonitides":ti,ab,kw OR "Pulmonary Inflammation*":ti,ab,kw OR "COVID-19":ti,ab,kw OR "COVID 19":ti,ab,kw OR "COVID19":ti,ab,kw OR "SARS Coronavirus 2 Infection*":ti,ab,kw OR "2019-nCoV":ti,ab,kw OR "2019 nCoV":ti,ab,kw OR "SARS-CoV-2":ti,ab,kw OR "SARS CoV 2":ti,ab,kw OR "Coronavirus":ti,ab,kw OR "Influenza*":ti,ab,kw OR "Grippe":ti,ab,kw OR "Flu":ti,ab,kw)) | 333 |
| Ebsco | ("Machine learning" OR "artificial intelligence" OR "deep learning" OR "prediction model*" ) AND ("Intratracheal Intubation*" OR "Intubation" OR "Endotracheal Intubation*" OR "Respiratory Insufficiency" OR "Respiratory Failure*" OR "Respiratory Depression" OR "Ventilatory Depression") AND ("Pneumonia*" OR "Lung Inflammation*" OR "Pneumonitis" OR "Pneumonitides" OR "Pulmonary Inflammation*" OR "COVID-19" OR "COVID 19" OR "SARS Coronavirus 2 Infection*" OR "2019-nCoV" OR "2019 nCoV" OR "SARS-CoV-2" OR "SARS CoV 2" OR "Coronavirus" OR "2019-nCoV" OR "2019 nCoV" OR "COVID19" OR "Influenza*" OR "Grippe" OR "Flu") | 345 |
| Google scholar | (machine learning) AND (pneumonia) AND ((intubation) OR (respiratory failure)) | 200 |
| Total |  | 2315 |

| 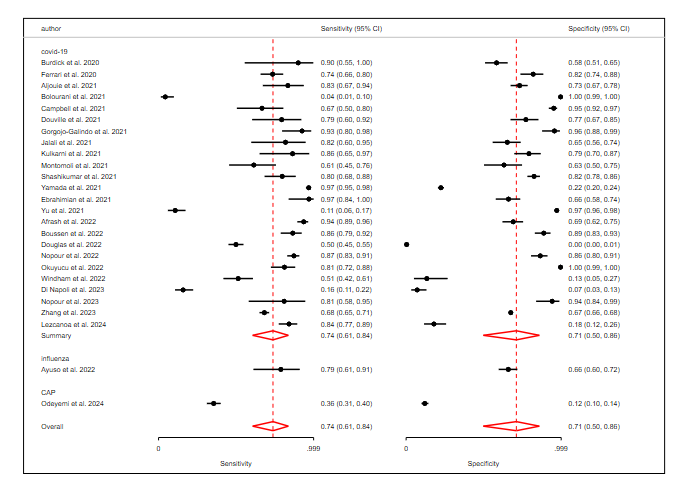 |
| --- |
| Supplementary figure 1. forest plot based on disease type. |


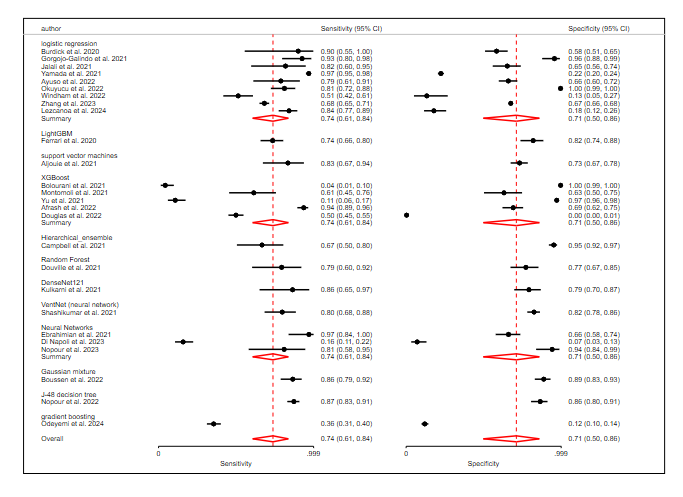


| Supplementary figure 2. forest plot based on model type. |
| --- |


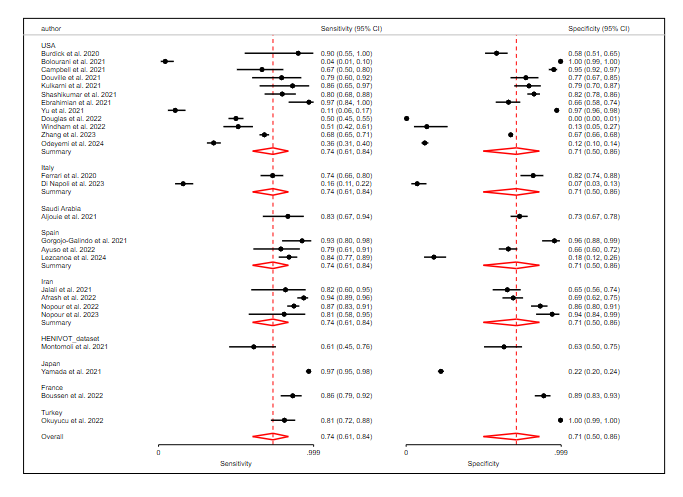


| Supplementary figure 3. forest plot based on country. |
| --- |
| 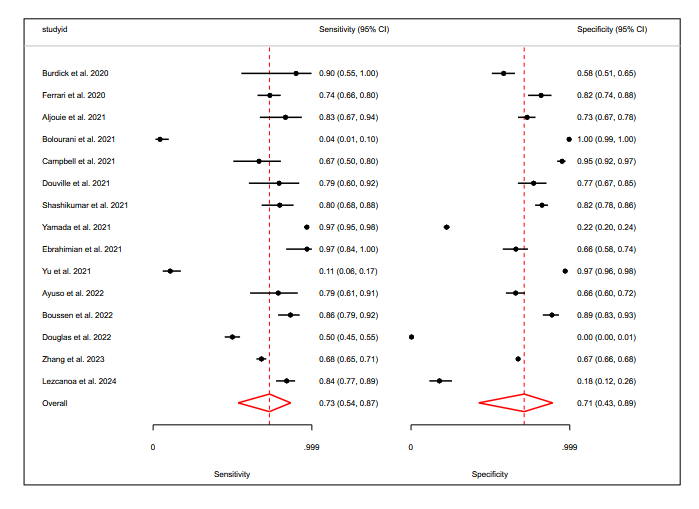 |
| Supplementary figure 4. forest plot based on Low-Risk of bias Studies. |
| 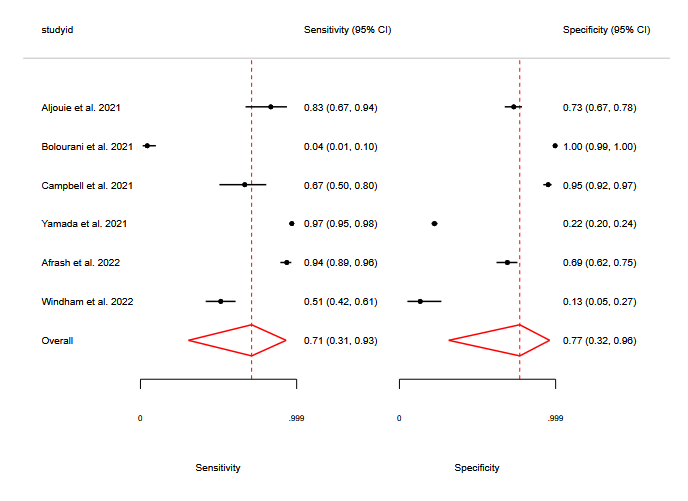 |
| Supplementary figure 5. forest plot based on studies that performed external validation. |
| 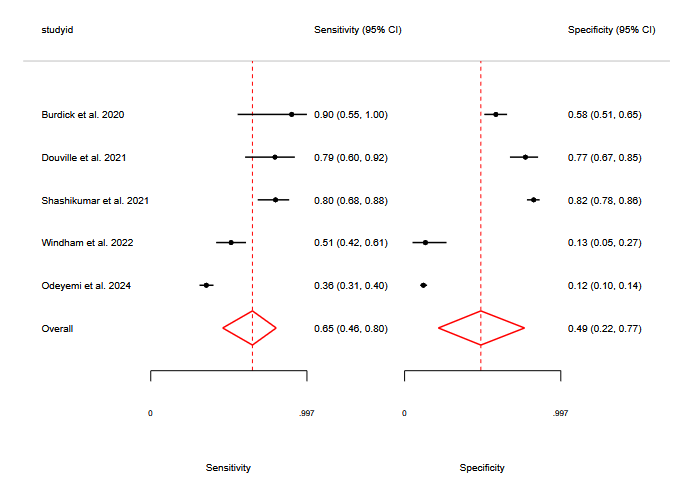 |
| Supplementary figure 6. forest plot based on studies predicted intubation within 24 hours. |
| 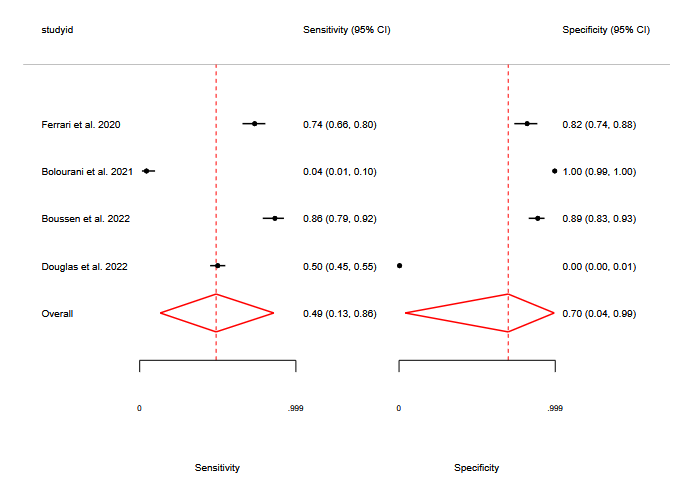 |
| Supplementary figure 7. forest plot based on studies predicted intubation within 48 hours. |
| 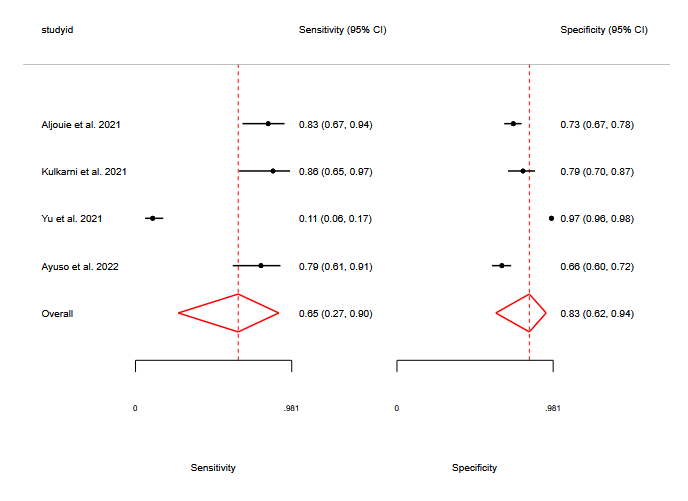 |
| Supplementary figure 8. forest plot based on studies predicted intubation within 72 hours. |
| 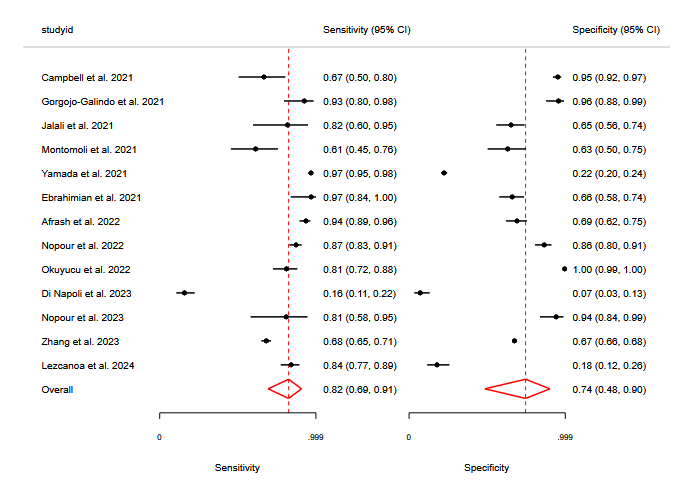 |
| Supplementary figure 9. forest plot based on studies predicted intubation during hospitalization period. |
| 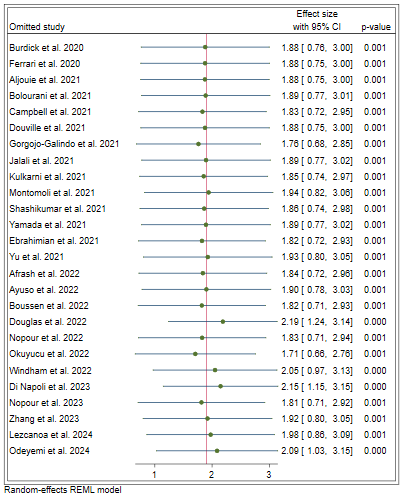 |
| Supplementary figure 10. The leave-one-out sensitivity analysis. |

| **supplementary table 3. The leave-one-out sensitivity analysis.** | | | | |
| --- | --- | --- | --- | --- |
| study_removed | Sensitivity | Specificity | I2_Sens | I2_Spec |
| Burdick et al. 2020 | 0.722 | 0.712 | 98.2 | 99.7 |
| Ferrari et al. 2020 | 0.729 | 0.702 | 98.15 | 99.71 |
| Aljouie et al. 2021 | 0.724 | 0.707 | 98.21 | 99.7 |
| Bolourani et al. 2021 | 0.757 | 0.67 | 97.53 | 99.66 |
| Campbell et al. 2021 | 0.732 | 0.69 | 98.21 | 99.7 |
| Douville et al. 2021 | 0.726 | 0.705 | 98.22 | 99.71 |
| Gorgojo-Galindo et al. 2021 | 0.717 | 0.69 | 98.16 | 99.7 |
| Jalali et al. 2021 | 0.725 | 0.71 | 98.22 | 99.7 |
| Kulkarni et al. 2021 | 0.723 | 0.704 | 98.21 | 99.71 |
| Montomoli et al. 2021 | 0.733 | 0.711 | 98.2 | 99.71 |
| Shashikumar et al. 2021 | 0.726 | 0.702 | 98.2 | 99.7 |
| Yamada et al. 2021 | 0.707 | 0.725 | 97.93 | 99.57 |
| Ebrahimian et al. 2021 | 0.714 | 0.709 | 98.11 | 99.7 |
| Yu et al. 2021 | 0.752 | 0.685 | 97.79 | 99.69 |
| Afrash et al. 2022 | 0.714 | 0.708 | 98.09 | 99.7 |
| Ayuso et al. 2022 | 0.727 | 0.71 | 98.22 | 99.7 |
| Boussen et al. 2022 | 0.722 | 0.698 | 98.18 | 99.7 |
| Douglas et al. 2022 | 0.737 | 0.754 | 97.83 | 99.59 |
| Nopour et al. 2022 | 0.721 | 0.699 | 98.1 | 99.7 |
| Okuyucu et al. 2022 | 0.725 | 0.671 | 98.18 | 99.66 |
| Windham et al. 2022 | 0.737 | 0.73 | 98.12 | 99.69 |
| Di Napoli et al. 2023 | 0.75 | 0.735 | 97.88 | 99.68 |
| Nopour et al. 2023 | 0.726 | 0.692 | 98.22 | 99.7 |
| Zhang et al. 2023 | 0.731 | 0.709 | 97.63 | 99.44 |
| Lezcanoa et al. 2024 | 0.723 | 0.727 | 98.16 | 99.69 |
| Odeyemi et al. 2024 | 0.742 | 0.731 | 97.77 | 99.66 |

| **Supplementary table 4. Meta-regression results** | | | | |
| --- | --- | --- | --- | --- |
| **Parameter** | **LRT χ²** | **p-value** | **I² (%)** | **95% CI for I²** |
| Pneumonia type | 1.59 | 0.45 | 0 | 0–100 |
| Model type | 0.92 | 0.63 | 0 | 0–100 |
| Feature set | 1.26 | 0.53 | 0 | 0–100 |
| Country | 1.79 | 0.41 | 0 | 0–100 |
| Overall bias | 0.00 | 1.00 | 0 | 0–100 |
| Study period | 0.69 | 0.71 | 0 | 0–100 |
| Validation type | 0.14 | 0.93 | 0 | 0–100 |
| Sample size | 1.97 | 0.37 | 0 | 0–100 |

| **Supplementary Table 5. Summary of findings for sensitivity and specificity with explicit GRADE downgrades by domain** | | | | | |
| --- | --- | --- | --- | --- | --- |
| **Outcome / Subgroup** | **Pooled Sensitivity (95% CI)** | **Pooled Specificity (95% CI)** | **AUROC (95% CI)** | **Certainty (GRADE)** | **Downgrades (Reason)** |
| **Overall meta-analysis** | 0.74 (0.61–0.84) | 0.71 (0.50–0.86) | 0.79 (0.75–0.82) | Moderate | −1 for inconsistency (I²=92%), −1 for publication bias |
| **COVID-19 subgroup** | 0.75 (0.61–0.85) | 0.74 (0.52–0.88) | 0.81 (0.77–0.84) | Moderate | −1 for inconsistency |
| **Logistic regression models** | 0.84 (0.72–0.91) | 0.66 (0.32–0.89) | 0.85 (0.81–0.88) | Moderate | −1 for imprecision |
| **XGBoost models** | 0.40 (0.10–0.80) | 0.72 (0.08–0.99) | 0.52 (0.47–0.56) | Low | −1 for inconsistency, −1 for imprecision |
| **Iran studies** | 0.89 (0.84–0.93) | 0.81 (0.66–0.90) | 0.93 (0.90–0.95) | Moderate | −1 for indirectness (limited region) |
| **USA studies** | 0.61 (0.37–0.80) | 0.67 (0.30–0.91) | 0.67 (0.62–0.71) | Low | −1 for inconsistency, −1 for imprecision |
| **Low risk of bias (PROBAST+AI)** | 0.73 (0.54–0.87) | 0.71 (0.43–0.89) | 0.78 (0.75–0.82) | Moderate | −1 for inconsistency |
| **Prediction within 24 h** | 0.65 (0.46–0.80) | 0.49 (0.22–0.77) | 0.73 (0.69 - 0.76) | Low | −1 for inconsistency (I²_sens=79.97%, I²_spec=96.92%), −1 for imprecision |
| **Prediction within 48 h** | 0.49 (0.13–0.86) | 0.70 (0.04–0.99) | 0.57 (0.53–0.62) | Low | −1 for inconsistency (I²_gen=76.42%), −1 for imprecision |
| **Prediction within 72 h** | 0.64 (0.27–0.90) | 0.83 (0.62–0.94) | 0.83 (0.80–0.86) | Moderate | −1 for inconsistency (I²_sens=89.75%, I²_spec=95.67%) |
| **Prediction during hospitalization** | 0.82 (0.69–0.91) | 0.74 (0.48–0.90) | 0.86 (0.83–0.89) | Moderate | −1 for inconsistency (I²_gen=91.96%) |
